# Association between cytokine profiles and lung injury in COVID-19 pneumonia

**DOI:** 10.1186/s12931-020-01465-2

**Published:** 2020-07-29

**Authors:** Li-Da Chen, Zhen-Yu Zhang, Xiao-Jie Wei, Yu-Qing Cai, Weng-Zhen Yao, Ming-Hui Wang, Qiu-Fen Huang, Xiao-Bin Zhang

**Affiliations:** 1 Department of Respiratory and Critical Care Medicine, Zhangzhou Affiliated Hospital of Fujian Medical University, Zhangzhou, Fujian Province China; 2 Department of Geriatrics, Zhongshan Hospital, Xiamen University; Teaching Hospital of Fujian Medical University, Xiamen, Fujian China; 3 Department of Pulmonary and Critical Care Medicine, Fujian Third People’s Hospital, Fuzhou, Fujian China; 4 Department of Pulmonary and Critical Care Medicine, Zhongshan Hospital, Xiamen University; Teaching Hospital of Fujian Medical University, No. 201, Hubin Nan Road, Siming District, Xiamen, Fujian Province 361004 People’s Republic of China

**Keywords:** Coronavirus disease 2019, Severe acute respiratory syndrome coronavirus 2, Cytokine, Lung injury, Pneumonia

## Abstract

**Background:**

Coronavirus disease 2019 (COVID-19) is a new respiratory and systemic disease caused by severe acute respiratory syndrome coronavirus 2 (SARS-CoV-2) infection. The purpose of the present study was to investigate the association between cytokine profiles and lung injury in COVID-19 pneumonia.

**Methods:**

This retrospective study was conducted in COVID-19 patients. Demographic characteristics, symptoms, signs, underlying diseases, and laboratory data were collected. The patients were divided into COVID-19 with pneumonia and without pneumonia. CT severity score and PaO_2_/FiO_2_ ratio were used to assess lung injury.

**Results:**

106 patients with 12 COVID-19 without pneumonia and 94 COVID-19 with pneumonia were included. Compared with COVID-19 without pneumonia, COVID-19 with pneumonia had significantly higher serum interleukin (IL)-2R, IL-6, and tumor necrosis factor (TNF)-α. Correlation analysis showed that CT severity score and PaO_2_/FiO_2_ were significantly correlated with age, presence of any coexisting disorder, lymphocyte count, procalcitonin, IL-2R, and IL-6. In multivariate analysis, log IL6 was the only independent explanatory variables for CT severity score (β = 0.397, *p* < 0.001) and PaO_2_/FiO_2_ (β = − 0.434, *p* = 0.003).

**Conclusions:**

Elevation of circulating cytokines was significantly associated with presence of pneumonia in COVID-19 and the severity of lung injury in COVID-19 pneumonia. Circulating IL-6 independently predicted the severity of lung injury in COVID-19 pneumonia.

## Backgrounds

In December, 2019, a cluster of patients with “unknown viral pneumonia” were reported in Wuhan, Hubei province, China. Then it was confirmed that the disease was caused by a novel coronavirus which was named severe acute respiratory syndrome coronavirus 2 (SARS-CoV-2). In March, the World Health Organization (WHO) declared that the outbreak of coronavirus disease 2019 (COVID-19) has become a global pandemic. Up to May 6, COVID-19 has spread to more than 200 countries with over 3600, 000 laboratory confirmed cases around the world and over 250, 000 death cases. The rapid spread of this disease around the world poses a severe threat to global health.

COVID-19 is a new respiratory and systemic illness with multiple organ damage, among which the lung is the main target organ. Post-mortem lung tissue of COVID-19 patients revealed extensive alveolar oedema, proteinaceous exudate, fibrin deposition, and immune cell infiltration [1]. Similar to other viral infection disease such as severe acute respiratory syndrome (SARS) and middle east respiratory syndrome (MERS), the cytokine storm was believed to be one of the major mechanisms which contribute to acute lung injury (ALI) and disease development [2, 3]. A previous study found that intensive care unit (ICU) patients with COVID-19 had higher plasma levels of interleukin (IL)-10, IL-2, IL-7, tumor necrosis factor (TNF)-α, IP-10, monocyte chemoattractant protein 1, Macrophage inflammatory protein 1A than those in non-ICU patients with COVID-19 [4]. Another study including 21 COVID-19 cases reported that severe cases had increased IL-2R, IL-6, IL-10, and TNF-α when compared to moderate cases [5]. However, there is no data evaluating the relationship between cytokine status and lung injury in COVID-19 pneumonia patients.

In the present retrospective study, we focused on the relationship between cytokine profiles and lung injury in COVID-19 pneumonia patients. First, we aimed to compare cytokine profiles between COVID-19 patients with pneumonia and without pneumonia. Second, we aimed to evaluate the relationship between cytokine profiles and lung injury assessed by computed tomographic (CT) findings and PaO_2_/FiO_2_ ratio in COVID-19 patients with pneumonia.

## Methods

### Patients

This was a retrospective observational study carried out in Optics Valley Branch of *Tongji Hospital.* Consecutive discharged patients in Optics Valley Branch of *Tongji Hospital* treated by Fujian Medical Team aiding Hubei province were enrolled in the study between January 28, 2020 and March 30, 2020. Inclusion criteria were as follows: 1. COVID-19 patients who had clinical symptoms were confirmed by positive SARS-CoV-2 real-time RT-PCR results. 2. Patients had completed laboratory data of cytokines. Patients with age less than 18 years old were excluded. This retrospective study was approved by the Ethics Committee of Zhongshan Hospital, Xiamen University. Informed consent was obtained from patients involved before data were collected retrospectively.

### Data collection

The medical records of all COVID-19 patients with positive SARS-CoV-2 real-time RT-PCR results were reviewed. The demographic data, comorbidities, clinical symptoms, signs, first time of laboratory findings during hospitalization, chest CT findings were collected. All data were checked by a team of trained physicians.

### Grouping criteria

COVID-19 patients were classified as mild cases, moderate cases, severe cases, and critical ill cases according to the guidelines for diagnosis and management of COVID-19 (7th edition, in Chinese) released by National Health Commission of China. Mild cases: the clinical symptoms are mild and no pneumonia manifestation can be found in imaging. Moderate cases: patients have symptoms such as fever and respiratory tract symptoms, etc., and pneumonia manifestation can be seen in imaging. Severe cases: adults who meet any of the following criteria: respiratory rate ≥ 30 breaths/min; SpO_2_ ≤ 93% at rest; PaO_2_/FiO_2_ ≤ 300. Patients with greater than 50% lesion progression wihin 24 to 48 hours in pulmonary imaging were also defined as severe cases. Critically ill cases: patients who meet any of the following criteria: occurrence of respiratory failure requiring mechanical ventilation; presence of shock; other organ failure that requires monitoring and treatment in the ICU. We further group mild cases as COVID-19 without pneumonia and moderate cases, severe cases, critical ill cases as COVID-19 with pneumonia.

### CT severity score

CT images were reviewed and scored independently by two respiratory and critical care physicians who were blinded to the clinical information in a consistent manner. CT severity score was evaluated based on the criteria as previously described [6, 7]. Briefly, each of the five lung lobes was assessed for percentage of the area involved. It was defined as none (0%), minimal (1–25%), mild (26–50%), moderate (51–75%), or severe (76–100%), with corresponded lobe score of 0, 1, 2, 3, 4, respectively. A CT severity score was calculated by summing the five lobe scores. The total score ranges from 0 to 20.

### Cytokine measurement

Blood samples were collected from the patients on admission or the second day after admission. Serum cytokines including IL-1β, IL-2R, IL-6, IL-8, IL-10, and TNF-α were measured using chemiluminescent immunoassay (CLIA) by Siemens Immulite 1000 analyzer according to the manufacturer’s instructions.

### Statistical analysis

Data analyses were performed using SPSS v 22.0 (SPSS Inc., Chicago, IL). Normally distributed, skewed, and categorical data were described using mean ± SD, median (interquartile range), and number (percentage), respectively. Student’s t test was conducted for two group comparison when variables were normally distributed; otherwise, the Mann–Whitney test was used. One-way ANOVA test was conducted for multiple group comparison when variables were normally distributed; otherwise, the Kruskal–Wallis H(K) test was used. Chi-square test or Fisher exact test were used to compare categorical variables. Spearman rank test was performed to test correlations between variables. In order to determine the independent predictors of lung injury, stepwise multiple linear regression analysis was performed. All descriptive data not in normal distribution were log-transformed before multivariate analysis. Statistical significance was determined as *p* < 0.05.

## Results

### Demographic data and clinical signs and symptoms

A total of 106 COVID-19 patients with 12 mild cases, 69 moderate cases, and 25 severe cases were included. They were further divided into two groups: COVID-19 without pneumonia (*n* = 12) and COVID-19 with pneumonia (*n* = 94). The baseline demographic and clinical data in different groups are presented in Table 1. COVID-19 patients with pneumonia were older and had a higher respiratory rate than COVID-19 patients without pneumonia. The presence of any coexisting disorder and symptom of fever were more common in COVID-19 patients with pneumonia than in those without pneumonia. The comparison of the data among the three groups was also performed. Age, the rate of any coexisting disorder, respiratory rate, and temperature increased, while SpO_2_ decreased significantly with the aggravation of the COVID-19 severity.

**Table 1:** The baseline demographic and clinical data in different groups

	Overall (*n* = 106)	COVID-19 without pneumonia/Mild (*n* = 12)	COVID-19 with pneumonia(*n* = 94)	*p* –values	Moderate (*n* = 69)	Severe (*n* = 25)	*p* –values
Age, years	52.75 ± 16.09	43.92 ± 13.73	53.87 ± 16.08	0.043	51.41 ± 15.77	60.68 ± 15.23	0.005
Male sex, n(%)	53 (50.0)	4 (33.3)	49 (52.1)	0.220	34 (49.3)	15 (60.0)	0.309
Coexisting disorder							
Any, n(%)	25 (23.6)	0 (0.0)	25 (26.6)	0.041	14 (20.3)	11 (44.0)	0.007
Hypertension, n(%)	17 (16.0)	0 (0.0)	17 (18.1)	0.108	9 (13.0)	8 (32.0)	0.028
Diabetes mellitus, n(%)	13 (12.3)	0 (0.0)	13 (13.8)	0.169	7 (10.1)	6 (24.0)	0.103
CHD, n (%)	2 (1.9)	0 (0.0)	2 (2.1)	1.000	1 (1.4)	1 (4.0)	0.578
COPD, n (%)	1 (0.9)	0 (0.0)	1 (1.1)	1.000	1 (1.4)	0 (0.0)	1.000
Symptoms							
Fever, n (%)	56 (52.8)	3 (25.0)	53 (56.4)	0.040	37 (53.6)	16 (64.0)	0.082
Cough, n (%)	52 (49.1)	5 (41.7)	47 (50.0)	0.587	34 (49.3)	13 (52.0)	0.839
Sputum production, n (%)	14 (13.2)	1 (8.3)	13 (13.8)	0.596	7 (10.1)	6 (24.0)	0.179
Fatigue, n (%)	20 (18.9)	0 (0.0)	20 (21.3)	0.076	13 (18.8)	7 (28.0)	0.124
Dyspnea, n (%)	7 (6.6)	0 (0.0)	7 (7.4)	0.328	3 (4.3)	4 (16.0)	0.097
Vital signs							
Temperature(°C)	36.50 (36.30–36.80)	36.50 (36.33–36.60)	36.50 (36.30–36.83)	0.270	36.50 (36.30–36.65)	36.90 (36.60–37.70)	0.001
Respiratory Rate	20.00 (20.00–20.00)	19.50 (19.00–20.00)	20.00 (20.00–20.25)	0.002	20.00 (20.00–20.00)	20.00 (20.00–22.00)	0.001
Heart Rate	89.89 ± 13.29	93.17 ± 14.48	89.47 ± 13.15	0.366	88.94 ± 13.94	90.92 ± 10.80	0.545
SpO_2_ (%)	98.00 (97.00–98.00)	98.00 (97.25–99.00)	98.00 (97.00–98.00)	0.174	98.00 (97.00–99.00)	96.00 (93.50–98.00)	< 0.001

*Abbreviation*: *COVID-19* coronavirus disease 2019, *CHD* coronary artery heart disease, *COPD* chronic obstructive pulmonary disease, *SpO*_*2*_ pulse oximeter oxygen saturation

### Laboratory data

The laboratory data in different groups are summarized in Table 2. D-dimer, fibrinogen, and high sensitive C reaction protein (hs-CRP) were higher in COVID-19 patients with pneumonia than in those without pneumonia. Laboratory data including blood routine, liver injury index, cardiac injury index, and other coagulation index were similar in both groups. The comparison of the data among the three groups showed a positive association between creatine kinase-MB, lactic dehydrogenase, D-dimer, fibrinogen, procalcitonin (PCT), hs-CRP and COVID-19 severity. A negative association between lymphocyte count, hemoglobin and COVID-19 severity was observed. There was a trend toward increased neutrophil count and activated partial thromboplastin time with the aggravation of the disease severity, but did not reach statistical significance.

**Table 2:** The laboratory data in different groups

	Overall (*n* = 106)	COVID-19 without pneumonia/Mild (*n* = 12)	COVID-19 with pneumonia(*n* = 94)	*p* –values	Moderate (*n* = 69)	Severe (*n* = 25)	*p* –values
White-cell count, ×10^9^/L	6.12 ± 2.29	5.80 ± 1.46	6.16 ± 2.38	0.618	6.03 ± 2.17	6.49 ± 2.92	0.615
Neutrophil count,× 10^9^/L	3.98 ± 2.14	3.44 ± 1.16	4.05 ± 2.23	0.357	3.77 ± 2.02	4.81 ± 2.64	0.075
Lymphocyte count, ×10^9^/L	1.50 ± 0.68	1.78 ± 0.54	1.46 ± 0.69	0.128	1.59 ± 0.67	1.12 ± 0.66	0.003
Hemoglobin, g/L	128.16 ± 21.07	132.17 ± 13.70	127.65 ± 21.83	0.487	130.71 ± 21.13	119.20 ± 21.91	0.049
PLT, ×10^9^/L	228.16 ± 91.20	215.08 ± 59.31	229.83 ± 94.60	0.600	234.59 ± 81.12	216.68 ± 125.64	0.615
ALT, U/L	24.50 (13.75–40.25)	24.50 (10.75–39.75)	24.50 (14.00–41.75)	0.654	25.00 (13.00–40.50)	24.00 (14.00–42.50)	0.883
AST, U/L	22.50 (15.00–32.25)	21.50 (15.50–27.50)	23.00 (15.25–35.25)	0.460	20.00 (14.50–52.00)	24.00 (15.50–46.00)	0.313
CK,U/L (*n* = 105)	76.09 ± 40.77	84.58 ± 34.67	74.99 ± 41.53	0.227	71.31 ± 36.51	85.00 ± 52.41	0.268
CK-MB, ng/mL (n = 105)	1.95 ± 4.49	0.67 ± 0.33	2.12 ± 4.75	0.083	1.08 ± 1.78	4.95 ± 8.14	< 0.001
LDH, U/L	193.00 (159.75–241.25)	180.00 (157.75–200.25)	196.50 (160.75–260.75)	0.214	190.00 (155.00–235.00)	239.00 (182.00–672.80)	0.007
APTT, s	37.65 (35.55–40.70)	36.90 (35.33–39.53)	37.90 (35.60–41.20)	0.440	38.30 (35.65–41.20)	36.50 (32.25–40.50)	0.085
PT, s	13.40 (12.80–13.93)	13.20 (12.90–13.68)	13.40 (12.83–14.00)	0.321	13.40 (12.90–14.00)	13.20 (11.85–14.30)	0.281
d-dimer, μg/mL	0.06 (0.05–0.08)	0.22 (0.22–0.22)	0.37 (0.22–0.81)	0.009	0.28 (0.22–0.64)	0.70 (0.32–1.50)	< 0.001
FIB, g/L	3.96 ± 1.60	3.05 ± 0.54	4.07 ± 1.65	< 0.001	3.78 ± 1.47	4.89 ± 1.87	0.001
Hs-CRP, mg/L (*n* = 98)	2.30 (0.68–8.40)	0.90 (0.33–1.83)(86)	2.45 (0.98–11.85)	0.008	2.25 (0.60–8.75)	3.95 (2.30–47.80)	0.002
PCT, ng/ml (*n* = 97)	0.06 (0.05–0.08	0.06 (0.05–0.07)	0.07 (0.05–0.09)	0.121	0.06 (0.05–0.08)(62)	0.08 (0.06–0.30)	0.001

*Abbreviation*: *IL* interleukin, *TNF* tumor necrosis factor, *Hs-CRP* high-sensitivity C-reactive protein, *CT* computed tomography, *PCT* procalcitonin, *COVID-19* coronavirus disease 2019, *PLT* platelet, *ALT* alanine aminotransferase, *AST* aspartate aminotransferase, *CK creatine kinase, LDH* lactic dehydrogenase, *APTT* activated partial thromboplastin time, *PT* prothrombin time, *FIB* fibrinogen

### Cytokine profiles between COVID-19 without and with pneumonia

Compared with COVID-19 without pneumonia, COVID-19 with pneumonia had significantly higher serum IL-2R, IL-6, and TNF-α. Serum cytokines including IL-2R, IL-6, IL-8, and TNF-α.were increased significantly with COVID-19 severity. The cytokine profiles in different groups are showed in the Table 3.

**Table 3:** The cytokine profile in different groups

	Overall (*n* = 106)	COVID-19 without pneumonia/Mild (*n* = 12)	COVID-19 with pneumonia(*n* = 94)	*p* –values	Moderate (*n* = 69)	Severe (*n* = 25)	*p* –values
IL-1β, pg/mL	5.00 (5.00–5.00)	5.00 (5.00–5.00)	5.00 (5.00–5.00)	0.726	5.00 (5.00–5.00)	5.00 (5.00–5.00)	0.829
IL-2R, U/mL	424.50 (268.75–692.75)	260.00 (229.25–358.50)	463.50 (281.50–716.75)	0.002	381.00 (266.00–631.00)	725.00 (471.00–968.50)	< 0.001
IL-6, pg/mL	3.48 (1.63–11.39)	1.85 (1.50–2.74)	3.86 (1.79–13.60)	0.016	2.90 (1.59–7.29)	17.05 (3.95–120.00)	< 0.001
IL-8, pg/mL	9.30 (6.25–12.48)	7.05 (6.15–11.18)	9.35 (6.45–12.95)	0.223	8.90 (5.80–12.05)	11.50 (9.05–18.00)	0.036
IL-10, pg/mL	5.00 (5.00–5.00)	5.00 (5.00–5.53)	5.00 (5.00–5.00)	0.849	5.00 (5.00–5.00)	5.00 (5.00–6.44)	0.198
TNF-α, pg/mL	7.60 (6.10–9.70)	6.35 (5.43–7.15)	7.95 (6.28–9.95)	0.016	7.50 (6.10–9.70)	8.70 (7.40–11.85)	0.007

*Abbreviation*: *IL* interleukin, *TNF* tumor necrosis factor, *COVID-19* coronavirus disease 2019

### Correlation analysis between lung injury and cytokine in COVID-19 with pneumonia

**Fig. 1:**
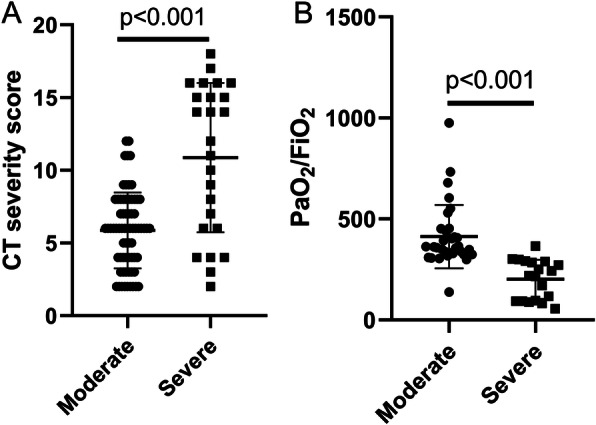
Comparisons of the CT severity score and PaO_2_/FiO_2_ between moderate group and severe group (**a**. CT severity score; **b**. PaO_2_/FiO_2_)

**Fig. 2:**
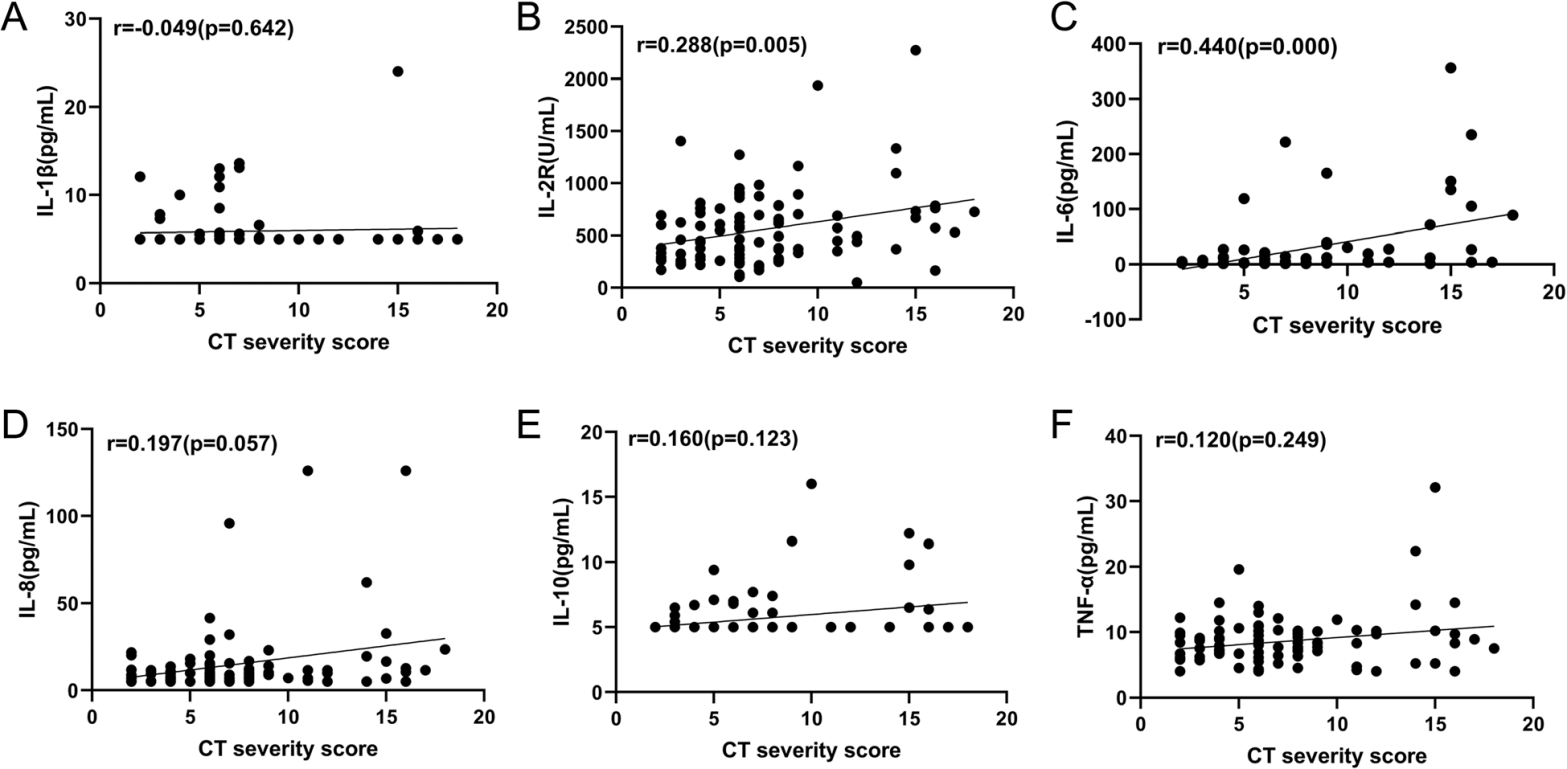
Correlations between cytokines and CT severity score (**a**. IL-1β; **b**. IL-2R; **c**. IL-6; **d**. IL-8; **e**. IL-10; **f**. TNF-α)

**Fig. 3:**
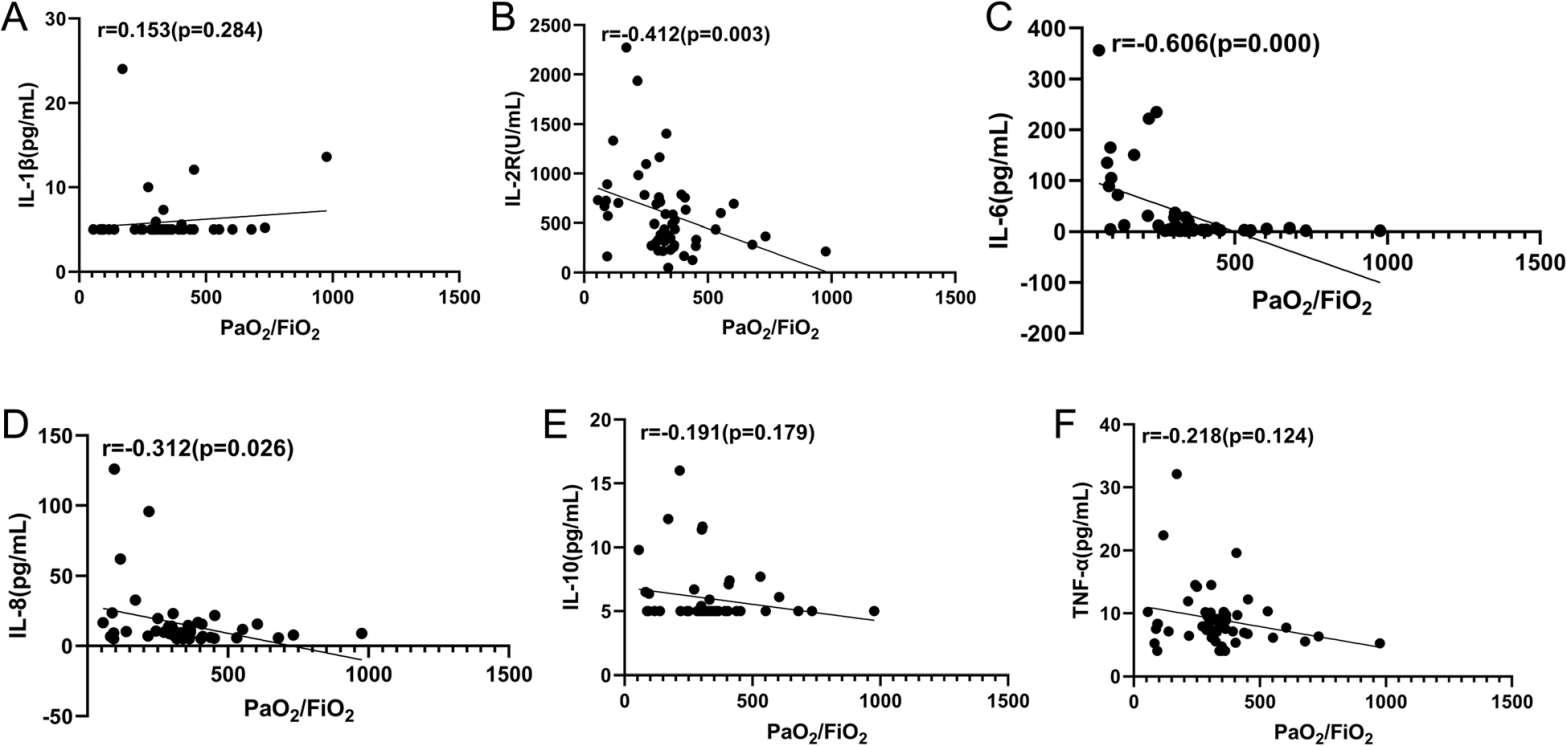
Correlations between cytokines and PaO_2_/FiO_2_ (**a**. IL-1β; **b**. IL-2R; **c**. IL-6; **d**. IL-8; **e**. IL-10; **f**. TNF-α)

We used CT severity score and PaO_2_/FiO_2_ ratio to assess lung injury in COVID-19 patients with pneumonia. Arterial blood gas analysis was not routinely performed in COVID-19 patients. Generally, it was more often performed in more severe COVID-19 patients. In total, 94 COVID-19 patients with pneumonia had the data of CT severity score, and 51 COVID-19 patients with pneumonia had the data of PaO_2_/FiO_2_ ratio. In COVID-19 patients with pneumonia, severe group had significantly higher CT severity score and lower PaO_2_/FiO_2_ than moderate group (Fig. 1). Correlation analysis between lung injury index and demographic data, laboratory data, as well as cytokines is presented in Table 4. CT severity score and PaO_2_/FiO_2_ were significantly correlated with age, presence of any coexisting disorder, lymphocyte count, PCT, IL-2R, and IL-6. IL-8 was significantly correlated with PaO_2_/FiO_2_, but not with CT severity score. The correlations between cytokines and CT severity score, PaO_2_/FiO_2_ are showed in Figs. 2 and 3, respectively.

**Table 4:** Spearman rank correlation coefficients between lung injury (CT severity score and PaO_2_/FiO_2_) and demographic data, laboratory data, and cytokines

	CT severity score(*n* = 94)		PaO_2_/FiO_2_(*n* = 51)	
	*r*	*p* –values	*r*	*p* –values
Age	0.283	0.006	−0.434	0.001
Sex	−0.095	0.361	0.177	0.213
Any coexisting disorder	0.208	0.045	−0.348	0.012
White-cell count	−0.063	0.546	0.114	0.426
Lymphocyte count	−0.419	< 0.001	0.565	< 0.001
Neutrophil count	0.058	0.580	−0.006	0.968
PCT	0.256	0.017	−0.424	0.002
Hs-CRP	0.352	0.001	−0.329	0.029
IL-1β	−0.049	0.642	0.153	0.284
IL-2R	0.288	0.005	−0.412	0.003
IL-6	0.440	< 0.001	−0.606	0000
IL-8	0.197	0.057	−0.312	0.026
IL-10	0.160	0.123	−0.191	0.179
TNF-a	0.120	0.249	−0.218	0.124
CT severity score	–	–	−0.460	0.001
PaO_2_/FiO_2_	−0.460	0.001	–	–

*Abbreviation*: *IL* interleukin, *TNF* tumor necrosis factor, *Hs-CRP* high-sensitivity C-reactive protein, *CT* computed tomography, *PCT* procalcitonin

### Predictors of lung injury in COVID with pneumonia

Stepwise multiple linear regression analyses were used to evaluate independent variables associated with CT severity score and PaO_2_/FiO_2_. The variables with statistical significance in Table 4 were taken as candidates for further stepwise multiple linear regression analyses. Independent variables including age, presence of any coexisting disorder, lymphocyte count, log IL-6, log IL-8, log IL-2R, log PCT and log hs-CRP were entered into the regression model, and CT severity score was taken as dependent variable. The results showed that only log IL-6 was included in the final model (β = 0.397, adjusted r^2^ = 0.147, *p* < 0.001). When PaO_2_/FiO_2_ was taken as dependent variable, the analysis identified the log IL-6 as the only independent explanatory variables for PaO_2_/FiO_2_ (β = − 0.434, adjusted r^2^ = 0.169, *p* = 0.003).

## Discussion

Main findings of this retrospective study are as follows: 1. COVID-19 patients with pneumonia had higher levels of IL-2R, IL-6, and TNF-α than COVID-19 patients without pneumonia. 2. Both IL-2R and IL-6 were statistically correlated with the severity of lung injury accessed by CT severity score and PaO_2_/FiO_2_ in COVID-19 patients with pneumonia.3. IL-6 was the independent predictor of the severity of lung injury in COVID patients with pneumonia after controlling for confounders. The findings of this study highlight the role of cytokines in mediating lung injury in COVID-19 pneumonia.

Cytokine storm is characterized by excessive inflammatory reaction in which proinflammatory cytokines are increasingly released in response to microbial infection. The process can result in tissue injury and an unfavorable prognosis in infectious disease [8]. This phenomenon has been noted in COVID-19 [9] as well as other coronavirus disease such as SARS [10] and MERS [11]. ALI/acute respiratory distress syndrome (ARDS) is a common consequence of a cytokine storm in systemic circulation and the lung alveolar environment [11]. As early as 2004, Wong et al. [12] found that SARS patients had marked elevation of Th1 cytokine interferon (IFN)-gamma, inflammatory cytokines IL-1, IL-6 and IL-12 for at least 2 weeks after disease onset. Another study focusing on the cytokine profiles in MERS patients found that the severe group had significantly higher serum levels of IL-6 and CXCL-10 than the mild group, which suggested that IL-6 and CXCL-10 were elevated in MERS patients who developed severe diseases [13].

Some researchers have noticed the cytokine responses associated with SARS-CoV-2 infection and investigated the cytokine profiles in COVID-19 patients. Huang et al. [4] found that ICU COVID-19 patients had higher plasma levels of cytokine profiles than those in non-ICU COVID-19 patients. Hou et al. [14] showed that lymphocytes were significantly decreased while cytokines including IL-8, TNF-α, IL-2R, IL-10 and IL-6 were significantly increased with increased severity of COVID-19. SARS-CoV-2 infection could result in injury to multiple organs leading to multiorgan failure. Previous studies mainly focused on the relationship between cytokines profiles and the severity of COVID-19, which was characterized by a respiratory and sys*temic* infectious disease. Lung was the main targeted organ during SARS-CoV-2 infection and ARDS was the most important cause of COVID-19 death. Therefore, we aimed to evaluate the role of cytokines in lung injury in COVID-19 patients and attempted to find out a potential therapeutic target for the management lung injury in COVID-19 pneumonia.

In this study, CT severity score and PaO_2_/FiO_2_ were used to evaluate extent of lung lesions and hypoxemia respectively. These two indexes were chosen based on the murray score [15], which is used to characterize the severity of lung injury. We revealed that COVID-19 patients with pneumonia had significantly higher levels of serum IL-2R, IL-6, and TNF-α than COVID-19 patients without pneumonia. This result indicated that the elevation of cytokines was significantly associated with presence of COVID-19 pneumonia. Then we further analyzed data of patients with COVID-19 pneumonia and found that both serum IL-2R and IL-6 were statistically correlated with the severity of lung injury. The findings suggested that cytokines play an important role in the lung injury in COVID-19 pneumonia. We noted that lymphocyte count was positively correlated with PaO_2_/FiO_2_ in COVID-19 patients with pneumonia. This was supported by a previous study, which showed that the lower lymphocyte count was commonly seen in COVID-19 patients and was significantly correlated with disease severity. The results of flow cytometry showed that the lower lymphocyte count in COVID-19 was largely attributed to the decrease in number of T lymphocytes including CD4+ T cells and CD8+ T cells [5]. Our results indicated that the COVID-19 pneumonia patients with significantly lower lymphocyte count should be closely monitored due to higher risk of respiratory failure and ARDS.

The results of the present study have some clinical implications. We found that IL-6 was the independent predictor of the severity of lung injury in COVID patients with pneumonia after controlling for confounders. The findings directly provide the evidence supporting the favorable outcomes of IL-6R blockers tocilizumab treatment in COVID-19 patients. Tocilizumab can specifically block IL-6 from binding to the soluble and membrane-bound IL-6R and inhibit signal transduction of inflammatory process [16]. It has been suggested as a treatment of COVID-19 [17]. A study included 25 severe COVID-19 patients who received tocilizumab therapy reported that tocilizumab was associated with dramatic decline in inflammatory markers, radiological improvement and reduced ventilatory support requirements [18]. Another study retrospectively analyzing the outcomes of tocilizumab treatment in 21 severe and critical COVID-19 patients showed that tocilizumab was associated with immediate improvement of the symptoms, hypoxygenmia, and CT opacity changes in most of the patients [19]. Our results, together with other study findings, suggest that IL-6 could serve as a useful marker to guide tocilizumab therapy in COVID-19. It also can be used to predict the efficacy of tocilizumab therapy in COVID-19. Targeted therapy based on IL-6 level may be helpful to alleviate lung injury in COVID-19 pneumonia and decrease mortality. SARS-CoV-2 infection can lead to injury of multiple organs, it will be interesting to explore whether IL-6 in systemic circulation mediates other organ or tissue injury in COVID-19. Future studies are needed to clarify this issue.

Several limitations of this study should be considered when interpreting results. Firstly, the sample size of the present study was relatively small, especially the number of patients in the group of COVID-19 without pneumonia was limited; thus statistical non-significance may occur because of insufficient power. Secondly, the study was a retrospective design, which might result in some biases (eg, unclear records, incomplete data). Thirdly, there was not any critical ill case in the present study, which might restrict the generalizability of the results to critical ill COVID-19 patients. Fourthly, only 6 cytokines which are assumed to play crucial roles in COVID-19 pneumonia were evaluated. Other cytokines, which may also play important roles in infectious diseases were not measured in the present study..

## Conclusions

Our study showed that elevation of circulating cytokines was significantly associated with presence of pneumonia in COVID-19 and the severity of lung injury in COVID-19 pneumonia. Circulating IL-6 independently predicted the severity of lung injury in COVID-19 pneumonia. The findings of our study could help to better understand the role of cytokines in COVID-19 associated lung injury and highlight a potential therapeutic target for the management lung injury in COVID-19 pneumonia.

## Data Availability

All data generated or analyzed during this study are included in this published article.

## Abbreviations

COVID-19: Coronavirus disease 2019
SARS-CoV-2: Severe acute respiratory syndrome coronavirus 2
CT: Computed tomography
IL: Interleukin
WHO: World Health Organization
SARS: Severe acute respiratory syndrome
MERS: Middle east respiratory syndrome
ICU: Intensive care unit
TNF: Tumor necrosis factor
hs-CRP: High-sensitivity C-reactive protein
PCT: Procalcitonin
ALI: Acute lung injury
ARDS: Acute respiratory distress syndrome
IFN: Interferon
